# Relevance of Adopting a Hybrid Strategy Mixing Single-Use and Reusable Ureteroscopes for Stones Management: An Economic Study to Support the Best Strategy

**DOI:** 10.3390/jcm10122593

**Published:** 2021-06-11

**Authors:** Fanny Monmousseau, Julien Ramillon, Sophie Dubnitskiy-Robin, Benjamin Faivre d’Arcier, Martine Le Verger, Tanguy Le Fol, Franck Bruyère, Emmanuel Rusch, Solène Brunet-Houdard, Benjamin Pradère

**Affiliations:** 1 Health-Economic Evaluation Unit, CHU de Tours-Bretonneau, 2 Boulevard Tonnellé, 37044 Tours, France; ju.ramillon@gmail.com (J.R.); S.DUBNITSKIY-ROBIN@chu-tours.fr (S.D.-R.); E.RUSCH@chu-tours.fr (E.R.); solene.brunet-houdard@chu-tours.fr (S.B.-H.); 2 EA 7505—Education Ethics Health, Faculty of Medicine, University of Tours, 2 Boulevard Tonnellé, 37044 Tours, France; 3 Inserm UMR1246 SPHERE, Universities of Nantes and Tours, CHU de Tours-Bretonneau, 2 Boulevard Tonnellé, 37044 Tours, France; 4 Department of Urology, CHU de Tours-Bretonneau, 2 Boulevard Tonnellé, 37044 Tours, France; B.FAIVREDARCIER@chu-tours.fr (B.F.d.); F.BRUYERE@chu-tours.fr (F.B.); benjaminpradere@gmail.com (B.P.); 5 Pharmacy, CHU de Tours-Trousseau, Avenue de la République, 37170 Chambray-les-Tours, France; M.LEVERGER@chu-tours.fr; 6 Biomedical Unit, CHU de Tours-Trousseau, Avenue de la République, 37170 Chambray-les-Tours, France; T.LEFOL@chu-tours.fr; 7 PRES Centre Val de Loire, University of Tours, 60 Rue du Plat d’Étain, 37000 Tours, France; 8 Comprehensive Cancer Center, Department of Urology, Medical University of Vienna, Spitalgasse 23, 1090 Wien, Austria

**Keywords:** single-use ureteroscopes, urolithiasis, stone, hybrid strategy, cost analysis, cut-off values, disposable, medical devices

## Abstract

Endoscopic procedures such as ureteroscopy (URS) have seen a recent increase in single-use devices. Despite all the advantages provided by disposable ureteroscopes (sURSs), their cost effectiveness remains questionable, leading most teams to use a hybrid strategy combining reusable (rURS) and disposable devices. Our study aimed to create an economic model that estimated the cut-off value of rURS procedures needed to support the profitability of a hybrid strategy (HS) for ureteroscopy. We used a budget impact analysis (BIA) model that estimated the financial impact of an HS compared to 100% sURS use. The model included hospital volume, sterilization costs and the private or public status of the institution. Although the hybrid strategy generally remains the best economic and clinical option, a predictive BIA model is recommended for the decision-making. We found that the minimal optimal proportion of rURS procedures in an HS was mainly impacted by the activity volume and overall number of sterilization procedures. Private and public institutions must consider these variables and models in order to adapt their HS and remain profitable.

## 1. Introduction

Single-use devices are rapidly developing in many medical specialties, including endourology [1]. Since 2015, single-use flexible ureteroscopes (sURSs) have been a major technological advancement in urolithiasis management. Their clinical performance and functional capabilities are comparable [2,3] to those of reusable ureteroscopes (rURSs). However, rURSs have disadvantages: they are fragile and prone to damage, and need to be reprocessed and maintained. Previously, the overall cost of an rURS (purchase, reprocessing, maintenance and repair) has been compared to the purchase cost of an sURS [4,5,6,7,8,9]. It was shown that hospitals’ volume of activity was an important factor of the final cost. Indeed, only low-volume centers could financially benefit from the use of sURS, with a higher overall cost of sURSs beyond 60–120 procedures depending on the sURS purchase price [10,11,12].

Therefore, it was proposed to only use sURSs for challenging procedures in which rURSs are at high risk of damage [9,13,14,15,16]. This hybrid strategy (HS) enables us to benefit from the advantages of sURSs (no reprocessing, availability and ease of use without breakage [7]) while reducing their financial impact. Nevertheless, the ratio between the two devices in an HS cannot be chosen randomly. As rURSs have fixed costs (purchase, reprocessing, maintenance), they need to be amortized. Therefore, the HS can only benefit if enough ureteroscopies are performed with the rURS to amortize the fixed costs and take advantage of economies of scale. For that reason, a minimal proportion of rURS procedures need to be defined as a “cut-off value” in the HS. This cut-off value can differ across various institutions according to their type (private or public), their activity volume and the total number of sterilization procedures due to the use of reusable endoscopes.

In this study, we seek to create a model that estimates the cut-off value of rURS procedures in an HS to support health care professionals in their choice of device strategy.

## 2. Methods

### 2.1. Analytic Framework

In a previous study, we proposed a budget impact model that estimated the 5-year financial impact of replacing all flexible fiberoptic rURSs by sURSs [17]. This impact was calculated for a public hospital where lithiasis surgery was a loss-making activity (with costs greater than revenue). We showed that completely switching to sURS increased this deficit.

In this study, the model was used to estimate the financial impact of an HS when the proportion of rURSs could continuously vary between 0% and 100%. For each proportion, the financial impact of the HS was then compared to the financial impact of switching to 100% sURS use. The model was adapted to consider the hospital volume, the sterilization costs, and the private or public status of the institution.

### 2.2. Hospital’s Activity Volume

The analysis of the data collected by the French Agency for Information on Hospital Care (AIHC) showed that the annual activity volume of the institutions (private and public) could vary between approximately 10 and 800 stone patients in 2019. The model was used on different values in this range to provide a cut-off value for each institution that corresponded to its activity volume.

### 2.3. Sterilization Costs

In order to consider the latest international recommendations for ureteroscope sterilization, we estimated the costs of low-temperature sterilization from a local micro-costing in our facility and from literature data [18]. Its total cost was estimated from the amortized cost of materials (Sterrad^®^ (ASP, Irvine, CA, USA), washer-disinfector), consumables and staff costs. The values used are reported in Table A1. The estimation of the amortized cost of sterilization equipment was based on the total number of overall endoscopes that were reprocessed in the hospital (i.e., ureteroscopes, bronchoscopes, cystoscopes, choledoscopes, etc.). The higher the number of endoscopes, the lower the cost of sterilization. We estimated that if less than 300 endoscopes were to be sterilized, the facilities were only equipped with one Sterrad^®^ and that at least two Sterrad^®^ units were required if more than 300 devices were to be sterilized (Table A1).

As the number of endoscopes to be sterilized depended on the practices of the institution, the sterilization cost of an rURS was estimated for different values. We assumed that the total number of endoscope sterilization procedures per year could represent 5 to 15 times the number of stone patients who may be treated by reusable ureteroscopes. This multiplying factor, ranging from 5 to 15, was called the “multiplying k-factor” in our model. The formula was as follows:

(1) $$k=\frac{\mathrm{total}\;\mathrm{number}\;\mathrm{of}\;\mathrm{endoscope}\;\mathrm{sterilization}\;\mathrm{procedures}}{\mathrm{number}\;\mathrm{of}\;\mathrm{stone}\;\mathrm{patients}}$$

### 2.4. Cost of rURS

The operating cost of a procedure performed with an rURS was calculated from a local micro-costing. It included the negotiated price of a fiberoptic rURS, the costs of the low-temperature sterilization, the price of maintenance contracts including repair services (for rURS and sterilization equipment) and the cost of quality control. As a result, an amortized rURS cost was estimated considering the distribution of fixed costs depending on the number of rURSs available in hospital and the number of ureteroscopies performed.

### 2.5. Public and Private Status

In France, 64% of ureteroscopies performed for urolithiasis in 2019 were carried out in private institutions. The costs and tariffs concerning diagnosis-related groups (DRGs) were notably different (Table A1). For example, the cost of the DRG for outpatient ureteroscopy in public institutions was equal to EUR 1256 (EUR 1621 in private). Additionally, the analysis of the AIHC data showed that the proportion of patients treated by ureteroscopy and their number over time varied between public and private hospitals (Table A1). Therefore, the results of the model were reported according to the type of institution (public or private).

### 2.6. Cut-Off Value Estimation

The hybrid strategy’s financial impact was estimated for different proportions of rURS between 0% and 100% according to the type of institution (public or private), the center volume and the overall number of endoscopes requiring sterilization per year. Then, it was compared to the financial impact of switching to 100% sURS use. The cut-off value was calculated as the minimum proportion of rURS procedures needed to ensure the hybrid strategy was financially preferable to 100% sURS use over a 5-year period.

## 3. Results

In the following, the results are reported by type of institution (public and private).

### 3.1. Public Institutions

**Figure 1:**
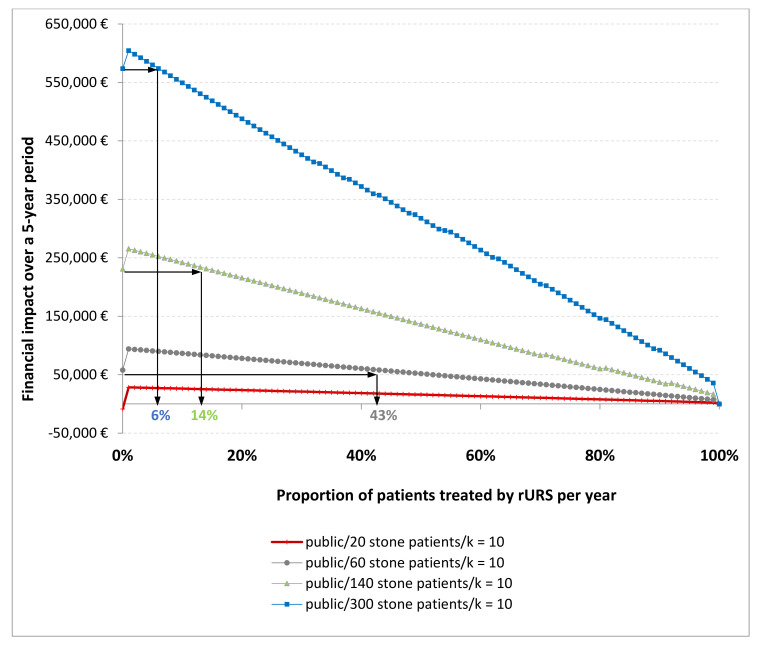
Graphical representation of the cut-off value for different types of facility. Note: The cut-off value can be read on the x-axis by projecting the value of the financial impact of switching completely to sURS use on the curve. For a public institution with 20 stone patients to be treated and 200 endoscope sterilization procedures (k = 10), the financial impact of switching to sURS use was negative (deficit reduction between costs and revenues) so any HS would not be profitable. For institutions with 60, 140 and 300 patients, the cut-off values were 43%, 14% and 6%, respectively, so the HS was more profitable than 100% sURS use.

For public hospitals, cut-off values are reported in Table 1 crossing the annual number of patients to be treated and the multiplying k-factor (to deduce the total number of endoscope sterilization procedures per year). Figure 1 gives the example of four hospitals belonging to the four categories identified.

The first profile defined very low-volume centers with a volume less than 30 patients per year. The deficit between costs and tariffs was reduced by completely switching to 100% sURS use. An HS was less profitable because the total cost of rURSs was borne by a small number of procedures. From a financial aspect, 100% sURS use was the best choice.

The second profile corresponded to centers that treated 35 to 100 patients. Depending on the k-factor value, the cut-off was estimated to be between 20% and 90%. This meant that they had to treat at least 2–9 out of 10 patients for the HS to result in a lower incremental cost than a complete switch to sURS.

The third profile corresponded to institutions that treated 100 to 200 patients. If at least 1–2 patients out of 10 were treated with an rURS, the HS was more profitable. This cut-off value was not heavily modified by the k-factor, as the number of endoscope sterilization procedures per year was high enough to amortize the fixed costs associated with the rURS.

The fourth profile was defined by high-volume centers with more than 200 patients per year. The cut-off value was less than 10%. This decreased with the volume of activity, and was independent of the k-factor. The volume of activity in endourology and in sterilization procedures per year was so high for these institutions that the fixed costs were quickly amortized. In this context, only a few procedures with rURSs were needed to make the HS more profitable (very low cut-off value) Therefore, these hospitals had significant freedom to implement an HS that was better most of the time than a 100% single-use strategy.

### 3.2. Private Institutions

Cut-off values for private institutions are reported in Table 2. The same four profiles were identified. The limit between each category was established for lower levels of activity volume due to the difference in costs and tariffs between public and private hospitals.

## 4. Discussion

Using a budget impact model, we explored the economic relevance of an HS mixing sURS and rURS use. We showed that the cut-off value between both devices must be predefined due to the high fixed costs related to rURS use. Our model allowed us to determine the minimum proportion of procedures to be performed with an rURS to support an HS from a financial perspective, compared to the use of 100% sURSs.

Our BIA-based model revealed the possibility of catering to the needs of surgeons as well as healthcare purchasers. It allowed us to accurately adapt the surgical strategy regarding medical device choice and plan the budget impact of the strategy used in advance. This model is highly relevant both clinically and economically, as it includes the most relevant variables required.

Although some argue that sterilization costs are not a solid benchmark [19], they have been shown to have a major economic impact. Compared to previous studies [10,11], we calculated the amortized cost of sterilization on the total number of endoscopes that are reprocessed (including ureteroscopes, bronchoscopes, cystoscopes and choledoscopes) and not only on the number of ureteroscopes. Omitting the overall cost of a sterilization unit is likely to bias the cost analysis and underestimate the real impact for the institution. Moreover, our cost analysis was based on the most up-to-date sterilization guidelines that recommend specific processes for all endoscopic devices from the same institution. Therefore, even if some institutions are still not equipped, our analysis allows them to get a preview of the generated cost resulting from the change to the new sterilization guidelines. Our analysis highlighted the importance of the caseload factor. Four institution profiles were described, revealing the need of completely different strategies. In institutions with a low-volume activity the hybrid strategy was not profitable and replacing all rURSs with sURSs was more beneficial. Indeed, the high costs associated with sterilization mainly impact small healthcare infrastructures. In hospitals with a high activity volume (more than 180 patients in private facilities or 220 patients in public facilities), the fixed costs associated with rURSs were amortized by the large volume of endoscopes. Only a small proportion of procedures performed with rURSs (less than 10%) was necessary to render the hybrid strategy more profitable. Hence, it would be easier for the high-volume centers to choose the ratio between sURS and rURS use in the implementation of an HS. Notably, these centers saw no economic benefit from switching to a 100% sURS strategy. Between these two extreme profiles, we showed that the cut-off value decreased with the activity volume. These results emphasize the importance of defining the case volume. Although many experts recommend the use of an HS based only on clinical cases [7,19,20,21], our study indicates that the number of patients treated can also be used to accurately implement the best hybrid strategy.

We demonstrated that budget impact analysis is a very powerful tool. In addition to estimating the financial impact of adopting a new procedure, it can be used to assess the relevance of a hybrid strategy combining two alternative procedures and to calculate cut-off values.

Budget impact analysis (BIA) is the final step in health technology assessment (HTA), and can allow health authorities to assess a new technology in terms of its safety and effectiveness, as well as its affordability for the health care system. It enables the accurate (re)allocation of financial resources in a given health system, either through the evaluation of new technologies or the re-evaluation of existing technologies. In the last decade, BIAs have been shown to have a promising role in decision-making due to the constant introduction of new premium-priced technologies on the market [22,23]. These new technologies push health care professionals to include the potential budget impact in their decision-making. Hence, health authorities and the main HTA institutions have expanded their guidelines to encompass BIAs [24,25]. Indeed, BIAs are of fundamental importance in budget allocation as well as in decisions regarding the pricing and utilization of new technologies. However, BIA studies have been criticized for not fulfilling the key characteristics for qualitative studies [26]. In our study, we were strongly committed to high methodological quality and low bias in order to enhance our BIA’s use among health authority decision-makers who will have to deal with single-use ureteroscopes. Our methodology even allowed us to undertake specific analyses in order to implement a hybrid strategy that included new and standard technologies.

We believe that our model and its external applicability are enhanced by its ease of use, since physicians or hospital managers only need to cross-reference two key parameters: the number of patients to be treated and a multiplying factor deduced from the total number of endoscope sterilization procedures.

We must acknowledge several limitations of this study. Firstly, the model used data describing the management of urolithiasis in French public hospitals, where patients are insured. Its applicability to foreign hospitals might be limited. The estimation of cut-off values was based on a prospective BIA including parameters whose values and evolution rate were based on retrospective national data and expert opinion. Among others, in future studies it could be interesting to apply the same BIA model to centers where bilateral URSs are performed [27], and where laparoscopic ureterolithotomy [28] is usually proposed. Indeed, these techniques could impact the number of URSs used in experienced centers. Finally, due to COVID-19 the recent changes in the prioritization of surgical management could affect the decision-making process regarding ureteroscopies for several years [29].

Nevertheless, these results provide the healthcare facilities with the first global preview of the budget impact of a hybrid strategy, and will help them to select the most appropriate one. Finally, we included only one type of sterilization in the analysis which could lead to a lack of external applicability for centers who have not yet embraced a global sterilization process for all endoscopes. However, this sterilization process is the most frequently reported, and its evaluation avoided the underestimation of the real cost related to this step.

## 5. Conclusions

We described a model based on budget impact analysis that allows healthcare purchasers and urologists to decide on the best strategy regarding the use of single-use and reusable ureteroscopes for the management of kidney stones. We showed that a hybrid strategy needed to be pre-planned and to consider the caseload and sterilization process. Although the hybrid strategy was generally the best economic and clinical option for the use of single-use and reusable devices, the use of a predictive model in advance is essential for the decision-making with this strategy.

## Figures and Tables

**Table 1:** Cut-off value for public hospitals in the case of the relevant hybrid strategy.

		Multiplying k-Factor to Obtain the Number of Endoscope Sterilization Procedures										
		5	6	7	8	9	10	11	12	13	14	15
Number of stone patients to be treated per year	10	NO	NO	NO	NO	NO	NO	NO	NO	NO	NO	NO
	15	NO	NO	NO	NO	NO	NO	NO	NO	NO	NO	NO
	20	NO	NO	NO	NO	NO	NO	NO	NO	NO	NO	NO
	30	NO	NO	91%	85%	89%	95%	NO	94%	NO	NO	NO
	35	90%	79%	72%	75%	79%	74%	86%	91%	85%	81%	77%
	40	72%	65%	67%	69%	72%	77%	77%	72%	68%	65%	63%
	50	52%	52%	60%	60%	62%	57%	53%	51%	49%	47%	46%
	60	44%	50%	49%	50%	45%	43%	41%	39%	38%	37%	36%
	70	39%	43%	42%	38%	36%	34%	33%	32%	31%	30%	29%
	80	36%	38%	34%	31%	29%	28%	27%	26%	26%	25%	25%
	90	33%	31%	28%	26%	25%	24%	23%	23%	22%	22%	22%
	100	31%	26%	24%	23%	22%	21%	20%	20%	20%	19%	19%
	120	22%	20%	19%	18%	17%	17%	16%	16%	16%	16%	15%
	140	18%	16%	15%	14%	14%	14%	13%	13%	13%	13%	13%
	160	14%	13%	13%	12%	12%	12%	11%	11%	11%	11%	11%
	180	12%	11%	11%	11%	10%	10%	10%	10%	10%	10%	10%
	200	11%	10%	10%	9%	9%	9%	9%	9%	9%	9%	9%
	220	9%	9%	8%	8%	8%	8%	8%	8%	8%	8%	8%
	230	9%	8%	8%	8%	8%	8%	8%	7%	7%	7%	7%
	300	6%	6%	6%	6%	6%	6%	6%	5%	5%	5%	5%
	400	4%	4%	4%	4%	4%	4%	4%	4%	4%	4%	4%
	500	3%	3%	3%	3%	3%	3%	3%	3%	3%	3%	3%
	600	3%	3%	2%	2%	2%	2%	2%	2%	2%	2%	2%
	700	2%	2%	2%	2%	2%	2%	2%	2%	2%	2%	2%
	800	2%	2%	2%	2%	2%	2%	2%	2%	2%	2%	2%

Note: For example, for a public institution with 80 stone patients to be treated per year and a total number of 640 endoscope sterilization procedures per year, k is equal to 8 (k = 640/80). The cut-off value is then read at the intersection of 80 and 8. In this case, the minimum percentage of rURSs in an HS, ensuring that the HS was financially preferable to 100% sURS use, is equal to 31%.

**Table 2:** Cut-off value for private hospitals in the case of the relevant hybrid strategy.

		Multiplying k-Factor to Obtain the Number of Endoscope Sterilization Procedures										
		5	6	7	8	9	10	11	12	13	14	15
Number of stone patients to be treated per year	11	NO	NO	NO	NO	NO	NO	NO	NO	NO	NO	NO
	15	NO	NO	NO	NO	NO	NO	NO	NO	NO	NO	NO
	20	NO	NO	NO	NO	NO	NO	NO	NO	96%	94%	92%
	30	88%	78%	71%	67%	71%	67%	65%	67%	69%	70%	68%
	35	69%	62%	57%	54%	52%	51%	52%	54%	58%	58%	60%
	40	56%	51%	48%	46%	47%	49%	50%	51%	50%	51%	50%
	50	41%	38%	36%	37%	40%	41%	42%	40%	39%	38%	37%
	60	32%	30%	32%	33%	33%	33%	32%	31%	30%	30%	29%
	70	28%	26%	28%	30%	28%	27%	26%	25%	25%	24%	24%
	80	23%	25%	25%	25%	23%	23%	22%	21%	21%	21%	20%
	90	21%	22%	22%	21%	20%	19%	19%	19%	18%	18%	18%
	100	20%	21%	19%	18%	17%	17%	17%	16%	16%	16%	16%
	120	16%	16%	15%	14%	14%	14%	13%	13%	13%	13%	13%
	140	14%	13%	12%	12%	11%	11%	11%	11%	11%	11%	11%
	160	11%	11%	10%	10%	10%	10%	9%	9%	9%	9%	9%
	180	10%	9%	9%	9%	8%	8%	8%	8%	8%	8%	8%
	200	8%	8%	8%	8%	7%	7%	7%	7%	7%	7%	7%
	220	7%	7%	7%	7%	7%	7%	7%	6%	6%	6%	6%
	230	7%	7%	7%	6%	6%	6%	6%	6%	6%	6%	6%
	300	5%	5%	5%	5%	5%	5%	5%	5%	4%	4%	4%
	400	3%	3%	3%	3%	3%	3%	3%	3%	3%	3%	3%
	500	3%	3%	3%	2%	2%	2%	2%	2%	2%	2%	2%
	600	2%	2%	2%	2%	2%	2%	2%	2%	2%	2%	2%
	700	2%	2%	2%	2%	2%	2%	2%	2%	2%	2%	2%
	800	1%	1%	1%	1%	1%	1%	1%	1%	1%	1%	1%

Note: This table is interpreted in the same way as Table 1.

## Data Availability

Not available.

## Appendix A

**Table A1:** Values and sources of the input parameters.

	Value for the First Year	Annual Evolution Rate
Urolithiasis rate	0.17% ^1^	+0.95% ^1^
SWL or ureteroscopy rate	0.22% ^2^	SWL: −5.51% ^1^ URS: 13.88% ^1^
Proportion of ureteroscopy compared to SWL	Public: 69.03%	+3.80% ^1^
	Private: 56.85% ^1^	+5.47% ^1,a^
Proportion of sURS compared to rURS	100.00%	0.00%
Assumptions		
Rate of rURS converted into SWL	1.00% ^2^	0.00%
Rate of SWL with anesthesia among SWL	10.00% ^2^	0.00%
Rate of SWL without anesthesia converted into sURS	25.00% ^2^	0.00%
Rate of SWL with anesthesia converted into sURS	100.00% ^2^	0.00%
Rate of outpatient activity for sURS strategy	53.66% ^3^	+10.00% ^1,b^
Rate of outpatient activity for rURS strategy	25.2% ^4^	+21% ^1,b^
DRG tariff for SWL (2020—11K08J)	Public: EUR 881 Private: EUR 438.64	0.00%
Anesthesia consultation tariff ^5^	EUR 25.00	0.00%
DRG tariff for ureteroscopy (2020—11C111)	Public: EUR 1867.30 Private: EUR 887.56	0.00%
Cost of the DRG for SWL without anesthesia ^6^	Public: EUR 1041.37 Private: EUR 724.35	0.0%
Cost of the DRG for SWL with anesthesia ^6^	Public: EUR 1188.01 Private: EUR 849.37	0.0%
Cost of the DRG for inpatient ureteroscopy ^6^	Public: EUR 2308.08 Private: EUR 1814.54	0.0%
Cost of the DRG for outpatient ureteroscopy ^6^	Public: EUR 1256.07 Private: EUR 1621.00	0.0%
Negotiated price of LithoVue^TM^ (2020) ^7^	EUR 834	0.00%
Cost of fiberoptic rURS	Purchase cost: EUR 1714.30	0.00%
	Maintenance: EUR 2000	0.00%
	Breakage rate: 10.0%	0.00%
	Quality check after breakage: EUR 66.8	0.0%
Simple-chamber washer-disinfector ^8^	Purchase cost: EUR 30,000	0.0%
	Maintenance: EUR 5000	0.0%
	Qualification: EUR 1500	0.0%
Double-chamber washer-disinfector ^8^	Purchase cost: EUR 50,000	0.0%
	Maintenance: EUR 7500	0.0%
	Qualification: EUR 1500	0.0%
Sterrad^®^/Small-capacity chamber ^8,c^	Purchase cost: EUR 75,000	0.0%
	Maintenance: EUR 7500	0.0%
	Qualification: EUR 8000	0.0%
Sterrad^®^/Large-capacity chamber ^8^	Purchase cost: EUR 100,000	0.0%
	Maintenance: EUR 10,000	0.0%
	Qualification: EUR 8000	0.0%
Sterilization—staff costs ^9^	EUR 45.9	0.0%
Sterilization—consumables costs ^10^	EUR 28.7	0.0%
Back-up benchtop ^11^	EUR 50,000	0.0%

^1^ Calculation from the authors from AIHC data; ^2^ According to expert opinion; ^3^ Calculation by authors using local data 2018 due to complete switch to sURS; ^4^ Calculation by authors using local data and AIHC data; ^5^ Tariff of the common classification of medical procedures of the French Health Insurance in 2020; ^6^ French national cost scale v2019 (2017 data adjusted for inflation); ^7^ Price obtained by the leading group purchasing cooperative network of French public hospitals (UniHA); ^8^ Data from local Biomedical Unit/amortization period: 10 years; ^9^ Data from local pharmacy; ^10^ Calculation from the authors from local data and [18]; ^11^ Data from local Biomedical Unit/amortization period: 20 years. ^a^ A decreasing annual growth rate over the time period was considered (27% lower than the previous year’s value). ^b^ A decreasing annual growth rate over the time period was considered (30% lower than the previous year’s value). ^c^ When less than 300 endoscopes were to be sterilized, the facilities were equipped with one Sterrad^®^ with a small-capacity chamber. Beyond that number, they were equipped with at least two Sterrad^®^ units with a large-capacity chamber, depending on the volume of endoscopes to be sterilized per year.

## References

[1] Moore B., Proietti S., Giusti G., Eisner B.H. (2019). Single-Use Ureteroscopes. Urol. Clin. N. Am..

[2] Schlager D., Obaid M.A., Hein S., Wilhelm K., Schönthaler M., Gratzke C., Miernik A., Schoeb D.S. (2020). Current Disposable Ureteroscopes: Performance and Limitations in a Standardized Kidney Model. J. Endourol..

[3] Eisel M., Strittmatter F., Ströbl S., Freymüller C., Pongratz T., Sroka R. (2020). Comparative investigation of reusable and single-use flexible endoscopes for urological interventions. Sci. Rep..

[4] Roberson D., Sperling C., Shah A., Ziemba J. (2020). Economic Considerations in the Management of Nephrolithiasis. Curr. Urol. Rep..

[5] Bayne D.B., Chi T.L. (2019). Assessing Cost-Effectiveness of New Technologies in Stone Management. Urol. Clin. N. Am..

[6] Talso M., Goumas I.K., Kamphuis G.M., Dragos L., Tefik T., Traxer O., Somani B.K. (2019). Reusable flexible ureterorenoscopes are more cost-effective than single-use scopes: Results of a systematic review from PETRA Uro-group. Transl. Androl. Urol..

[7] Marchini G.S., Torricelli F.C., Batagello C.A., Monga M., Vicentini F.C., Danilovic A., Srougi M., Nahas W.C., Mazzucchi E., Marchini G.S. (2019). A comprehensive literature-based equation to compare cost-effectiveness of a flexible ureteroscopy program with single-use versus reusable devices. Int. Braz. J. Urol..

[8] Taguchi K., Usawachintachit M., Tzou D.T., Sherer B.A., Metzler I., Isaacson D., Stoller M.L., Chi T. (2018). Micro-Costing Analysis Demonstrates Comparable Costs for LithoVue Compared to Reusable Flexible Fiberoptic Ureteroscopes. J. Endourol..

[9] Ozimek T., Schneider M.H., Hupe M.C., Wiessmeyer J.R., Cordes J., Chlosta P.L., Merseburger A.S., Kramer M.W. (2017). Retrospective Cost Analysis of a Single-Center Reusable Flexible Ureterorenoscopy Program: A Comparative Cost Simulation of Disposable fURS as an Alternative. J. Endourol..

[10] Martin C.J., McAdams S.B., Abdul-Muhsin H., Lim V.M., Nunez-Nateras R., Tyson M.D., Humphreys M.R. (2017). The Economic Implications of a Reusable Flexible Digital Ureteroscope: A Cost-Benefit Analysis. J. Urol..

[11] Mager R., Kurosch M., Höfner T., Frees S., Haferkamp A., Neisius A. (2018). Clinical outcomes and costs of reusable and single-use flexible ureterorenoscopes: A prospective cohort study. Urolithiasis.

[12] Al-Balushi K., Martin N., Loubon H., Baboudjian M., Michel F., Sichez P.-C., Martin T., Di-Crocco E., Gaillet S., Delaporte V. (2019). Comparative medico-economic study of reusable vs. Single-use flexible ureteroscopes. Int. Urol. Nephrol..

[13] Doizi S., Kamphuis G., Giusti G., Andreassen K.H., Knoll T., Osther P.J., Scoffone C., Pérez-Fentes D., Proietti S., Wiseman O. (2017). First clinical evaluation of a new single-use flexible ureteroscope (LithoVue^TM^): A European prospective multicentric feasibility study. World J. Urol..

[14] Wilson M., Warncke J., Donalisio da Silva R., Gustafson D., Nogueira L., Kim F. (2018). Pd53-03 cost analysis of utilization of disposable flexible ureteroscopes in high risk for breakage cases. J. Urol..

[15] Scotland K.B., Chan J.Y.H., Chew B.H. (2019). Single-Use Flexible Ureteroscopes: How Do They Compare with Reusable Ureteroscopes?. J. Endourol..

[16] Bahaee J., Plott J., Ghani K.R. (2021). Single-use flexible ureteroscopes: How to choose and what is around the corner?. Curr. Opin. Urol..

[17] Dubnitskiy-Robin S., Pradère B., Faivre d’Arcier B., Watt S., Le Fol T., Bruyère F., Rusch E., Monmousseau F., Brunet-Houdard S. (2020). Switching to Single-use Flexible Ureteroscopes for Stones Management: Financial Impact and Solutions to Reduce the Cost Over a 5-Year Period. Urology.

[18] Allainmat-Lemercier A., Taurin S., Mehault L., Hamon L. Coût de la Prise en Charge en Stérilisation des Endoscopes Souples au CHU de Rennes. Sterilisation Centrale 2017. https://docplayer.fr/47679058-Mots-cles-endoscope-souple-sterilisation-cout.html.

[19] Ventimiglia E., Godínez A.J., Traxer O., Somani B.K. (2020). Cost comparison of single-use versus reusable flexible ureteroscope: A systematic review. Turk. J. Urol..

[20] Ventimiglia E., Somani B.K., Traxer O. (2020). Flexible ureteroscopy: Reuse? Or is single use the new direction?. Curr. Opin. Urol..

[21] Li Y., Chen J., Zhu Z., Zeng H., Zeng F., Chen Z., Yang Z., Cui Y., Chen H., Li Y. (2021). Comparison of single-use and reusable flexible ureteroscope for renal stone management: A pooled analysis of 772 patients. Transl. Androl. Urol..

[22] Godman B., Acurcio F.A., Júnior A.A.G., Alvarez-Madrazo S., Aryani M.Y.F., Bishop I., Campbell S., Eriksson I., Finlayson A.E., Fürst J. (2014). Initiatives among authorities to improve the quality and efficiency of prescribing and the implications. J. Pharm. Care Health Syst..

[23] Godman Brian Malmström R.E., Diogene E., Gray A., Jayathissa S., Timoney A., Acurcio F., Alkan A., Brzezinska A., Bucsics A., Campbell S.M. (2015). Are new models needed to optimize the utilization of new medicines to sustain healthcare systems?. Expert Rev. Clin. Pharmacol..

[24] Mauskopf J.A., Earnshaw S., Mullins C.D. (2015). Budget impact analysis: Review of the state of the art. Expert Rev. Pharm. Outcomes Res..

[25] Garattini L., van de Vooren K. (2011). Budget impact analysis in economic evaluation: A proposal for a clearer definition. Eur. J. Health Econ. HEPAC Health Econ. Prev. Care.

[26] Faleiros D.R., Álvares J., Almeida A.M., de Araújo V.E., Andrade E.I.G., Godman B.B., Acurcio F.A., Guerra Júnior A.A. (2016). Budget impact analysis of medicines: Updated systematic review and implications. Expert Rev. Pharm. Outcomes Res..

[27] Li J., Chang X., Wang Y., Han Z. (2020). Laparoscopic ureterolithotomy versus ureteroscopic laser lithotripsy for large proximal ureteral stones: A systematic review and meta-analysis. Minerva Urol. E Nefrol. Ital. J. Urol. Nephrol..

[28] Ulker V., Cakmak O., Yucel C., Can E., Celik O., Ilbey Y.O. (2019). The efficacy and safety of bilateral same-session ureteroscopy with holmium laser lithotripsy in the treatment of bilateral ureteral stones. Minerva Urol. E Nefrol. Ital. J. Urol. Nephrol..

[29] Amparore D., Campi R., Checcucci E., Sessa F., Pecoraro A., Minervini A., Fiori C., Ficarra V., Novara G., Serni S. (2020). Forecasting the Future of Urology Practice: A Comprehensive Review of the Recommendations by International and European Associations on Priority Procedures During the COVID-19 Pandemic. Eur. Urol. Focus.

